# Establishment of a Model of Renal Impairment with Mild Renal Insufficiency Associated with Atrial Fibrillation in Canines

**DOI:** 10.1371/journal.pone.0105974

**Published:** 2014-08-26

**Authors:** Zhuo Liang, Li-feng Liu, Xin-pei Chen, Xiang-min Shi, Hong-yang Guo, Kun Lin, Jian-ping Guo, Zhao-liang Shan, Yu-tang Wang

**Affiliations:** 1 Department of Geriatric Cardiology, Chinese PLA General Hospital, Beijing, China; 2 Department of Cardiology, Chinese PLA General Hospital, Beijing, China; The University of Manchester, United Kingdom

## Abstract

**Background:**

Chronic kidney disease and occurrence of atrial fibrillation (AF) are closely related. No studies have examined whether renal impairment (RI) without severe renal dysfunction is associated with the occurrence of AF.

**Methods:**

Unilateral RI with mild renal insufficiency was induced in beagles by embolization of small branches of the renal artery in the left kidney for 2 weeks using gelatin sponge granules in the model group (n = 5). The sham group (n = 5) underwent the same procedure, except for embolization. Parameters associated with RI and renal function were tested, cardiac electrophysiological parameters, blood pressure, left ventricular pressure, and AF vulnerability were investigated. The activity of the sympathetic nervous system, renin-angiotensin-aldosterone system, inflammation, and oxidative stress were measured. Histological studies associated with atrial interstitial fibrosis were performed.

**Results:**

Embolization of small branches of the renal artery in the left kidney led to ischemic RI with mild renal insufficiency. The following changes occurred after embolization. Heart rate and P wave duration were increased. Blood pressure and left ventricular systolic pressure were elevated. The atrial effective refractory period and antegrade Wenckebach point were shortened. Episodes and duration of AF, as well as atrial and ventricular rate during AF were increased in the model group. Plasma levels of norepinephrine, renin, and aldosterone were increased, angiotensin II and aldosterone levels in atrial tissue were elevated, and atrial interstitial fibrosis was enhanced after 2 weeks of embolization in the model group.

**Conclusions:**

We successfully established a model of RI with mild renal insufficiency in a large animal. We found that RI with mild renal insufficiency was associated with AF in this model.

## Introduction

The prevalence of atrial fibrillation (AF) in the general population is 1% [1] A recent meta-analysis showed that the prevalence of AF in end-stage renal disease patients was 11.6% [2]. The Chronic Renal Insufficiency Cohort study suggested that the prevalence of AF is 2–3-fold higher in patients with mild-to-moderate chronic kidney disease (CKD) than in the general population [3]. Concomitant CKD increases the recurrence of AF after catheter ablation of AF [4]. Renal dysfunction is also associated with an increased risk of stroke and mortality in patients with AF [5]. Therefore, exploring the inherent pathogenic mechanisms responsible for the development of AF among CKD patients and identifying effective therapeutic targets are urgent. However, few studies on animals have investigated these pathogenic mechanisms because of a lack of an appropriate animal model.

The remnant kidney model has been the most well studied model of CKD [6]. Studies have used a range of remnant kidney models, from 1/2 to 15/16 nephrectomy. Removal of tissue is generally accomplished by surgery or infarction accomplished by ligation of renal arteries. The remnant kidney model is more focused on reduced renal function, irrespective of the primary renal impairment in CKD. Most nephrons are directly removed by surgery, not injured and retained in the body. Additionally, only the remnant nephrons become impaired in the long-term, which takes several months to years, resulting from glomerular hyperperfusion, hyperfiltration, hypertension, and other factors induced by renal dysfunction. The more remnant nephrons remain, the longer it takes for impairment of remnant nephrons. Ligation of renal arteries can lead to complete infarction, with damage to afferent and efferent nerves in the adventitia of the renal artery and area of the renal hilus. The process involved in creating the remnant kidney model causes severe trauma, which leads to a high mortality of animals, and is also complicated and time-consuming. CKD is accompanied by ischemic renal impairment (RI) and renal dysfunction. Multiple acquired factors induced by severe renal dysfunction in the remnant kidney model could account for the high prevalence of AF (e.g., hypervolemia, acidosis, hypertension, and electrolyte disturbance) [7]. The above-mentioned factors may affect the reliability of research when examining the inherent pathogenic mechanisms responsible for the development of AF among CKD patients. There is no appropriate animal model for ischemic RI and without severe renal dysfunction for examining the relationship between CKD and AF.

Therefore, in this study, we established a model of unilateral RI with mild renal insufficiency in canines. We examined whether renal impairment without severe renal dysfunction was associated with the occurrence of AF.

## Materials and Methods

### Ethics Statement

This study was carried out in strict accordance with the recommendations in the Guide for the Care and Use of Laboratory Animals of the National Institutes of Health (Publication No. 85-23, revised 1996). The protocol was approved by the Institutional Animal Care and Use Committee of the Chinese PLA General Hospital.

### Experimental Model for RI

The experimental animals included 10 healthy, 4–5-year-old beagles weighing 10–12 kg. All dogs were anesthetized with intravenous sodium pentobarbital (20 mg/kg) and were intubated using an endotracheal tube and mechanical ventilation. Heart rate and rhythm were monitored by a continuous 3-lead electrocardiogram. A 6F sheath was placed in the right femoral artery. Systolic blood pressure (SBP) and diastolic blood pressure (DBP) were monitored via the sheath using an invasive blood pressure (BP) monitor. A bolus of heparin (4000 IU) was administrated through the sheath to prevent thromboembolism. A pigtail catheter was introduced into the left ventricle (LV) through the arterial sheath to detect LV systolic pressure (LVSP) and LV end-diastolic pressure (LVEDP). A 5F multifunction catheter was introduced through the arterial sheath and renal artery angiography was performed under fluoroscopy. Following renal artery angiography, RI was induced in five dogs by transcatheter embolization of small branches of the left renal artery using gelatin sponge granules (diameter ∼50 µm), whereas the main renal artery or sub-segment renal artery was kept fluent.

### Electrophysiological Examinations

The right femoral vein was cannulated for catheter insertion. The tip of a multielectrode catheter was placed on the lateral right atrium to record right atrial potentials and to induce rapid atrial pacing. A train of eight basic stimuli (S1, pulse duration 1 ms) at twice the diastolic pacing threshold was followed by an extra stimulus (S2). The atrial effective refractory period (AERP) was defined as the longest S1S2 interval that failed to elicit a propagated atrial response. The AERP was measured at basic pacing cycle lengths of 300 ms and 240 ms, and the S1–S2 intervals were decreased from 200 ms to refractoriness by decrements of 5 ms (LEAD-7000, multi-channel physiology recorder; Sichuan Jinjiang Electronic Science and Technology Co., Ltd, Sichuan, China). The longest cycle length of atrial pacing causing second-degree atrioventricular nodal block (antegrade Wenckebach point) was determined. After the AERP and antegrade Wenckebach point were determined, rapid atrial pacing (basic cycle length, 60 ms) for 30 minutes was delivered (DF-5A, heart stimulator; Suzhou Dongfang Electronic Instruments Plant, Jiangsu, China) and then AERP was determined again. After the above examinations, 10 times of rapid atrial pacing were performed (60 ms of basic cycle length, 10 s in duration, four-fold threshold current) to induce AF. AF was defined as irregular atrial rates (cycle length, <200 ms; duration, >5 seconds) with irregular atrioventricular conduction. AF inductibility was defined as (the relative ratio of successful induction frequency to total frequency of pacing in each group) ×100%. All AA- and RR-intervals during AF were calculated to determine the mean atrial and ventricular rates during AF.

### Plasma Measurements and Urinalysis

Blood samples were collected from the femoral vein into tubes containing EDTA, and immediately centrifuged at 2310×g for 10 minutes at 4°C, and then finally stored at −80°C until further assay. Levels of norepinephrine, renin, aldosterone, high-sensitivity C-reactive protein (hs-CRP), malondialdehyde, creatinine, urea nitrogen, lactic dehydrogenase, and lactic acid in plasma and creatinine in urine were examined by ELISA (Wuhan Beinglay Biotech Co., Ltd., Hubei, China). Sodium and potassium concentrations in plasma were examined by an Electrolyte Analyzer (HC-9883, Shenzheng Histrong Medical Equipment Co., Ltd., Shenzheng, China).

### Histologic Studies

Left atria were carefully removed. Part of atrial tissue was fixed in 10% phosphate-buffered formalin, and embedded in paraffin. Deparaffined sections (5 µm thickness) were stained with Masson trichrome. Connective tissue was differentiated on the basis of its color and expressed as a percentage of the reference tissue area using Image-Pro Plus 4.5. In each atrium, 3 images with a magnification of ×400 were analyzed and averaged. Part of atrial tissue was stored at −80°C until further assay. Levels of angiotensin II and aldosterone in atrial tissue were examined by ELISA (Wuhan Beinglay Biotech Co., Ltd., Hubei, China).

### Experimental Design

Dogs were divided into two groups: the model group (n = 5) and the sham group (n = 5). At baseline, an electrocardiogram, BP, and LV function were monitored. Electrophysiological examinations were performed. Plasma parameters associated with RI, renal function, the activity of the sympathetic nervous system (SNS), renin-angiotensin-aldosterone system (RAAS), inflammation, and oxidative stress were measured. After these measurements, RI was induced in the model group. In the sham group, normal saline was injected into the renal artery through a multifunction catheter after renal artery angiography as a sham procedure. After 2 weeks of feeding, the same parameters measured at baseline were measured again. Creatinine clearance (CCr) was determined by 30-min endogenous creatinine clearance method [8]. Dogs were then sacrificed humanely by an intravenous overdose of thiopental (2 g). Kidneys were removed for hematoxylin and eosin (HE) staining and morphological analysis. Hearts were removed for histologic studies.

### Statistical Analysis

Values are shown as mean ± SD. For repeated-measures comparisons with the same baseline, repeated-measures 2-way ANOVA was used followed by the Dunnet test to compare individual mean difference if ANOVA was significant. Unpaired t tests were used to compare differences of angiotensin II, aldosterone and interstitial fibrosis in atrial tissue and CCr between sham and model groups. The chi-square test was used to compare the AF induction rate. P≤0.05 was considered statistically significant.

## Results

### Model of RI with Mild Renal Insufficiency

**Figure 1:**
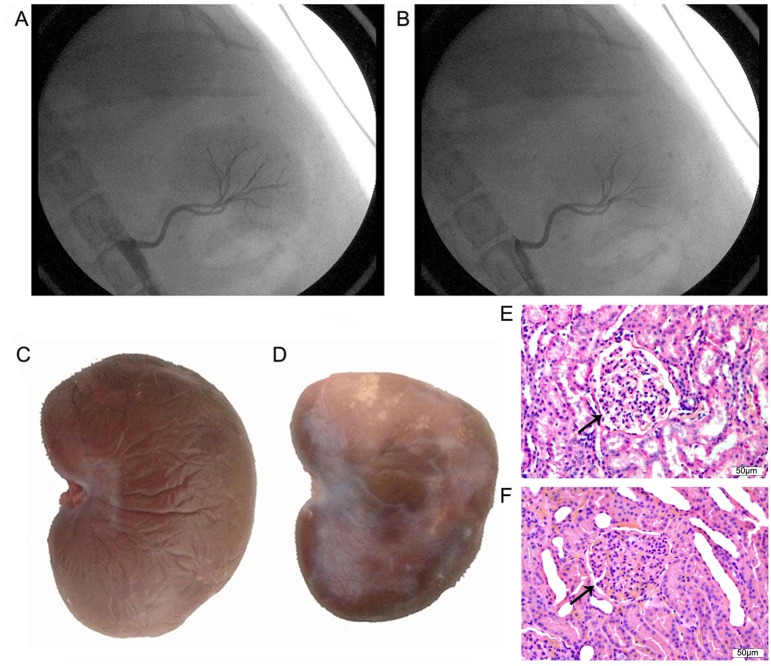
Images and morphological analysis of the left kidney. Images of left renal artery angiography before transcatheter embolization (A) and after transcatheter embolization (B) in the model group. Small renal artery branches were occluded, whereas the main renal artery or sub-segment renal artery remained fluent in the model group. Gross appearance of the left kidney after 2 weeks of interventional operation in the sham group (C) and the model group (D). The left kidney in the model group became pale and had atrophy and infarction. Images of HE staining of the left kidney after 2 weeks of interventional operation in the sham (E) and model (F) groups. Glomeruli were severely damaged in the model group. Arrows show a glomerulus in the sham and model groups.

Figure 1A shows representative images of left renal artery angiography before transcatheter embolization in the model group. Small renal artery branches were occluded after transcatheter embolization, whereas the main renal artery or sub-segment renal artery remained fluent (Figure 1B). Figure 1C shows a representative gross appearance of the left kidney in the sham group. After 2 weeks of embolization in the model group, the left kidney became pale due to ischemic impairment, and had atrophy and infarction (Figure 1D). Figure 1E shows representative images of HE staining of the left kidney in the sham group. Glomeruli were severely damaged in the model group (Figure 1F), indicating that the vast majority of nephrons had lost their function. Table 1 shows some of the parameters associated with renal impairment and renal function. Lactic dehydrogenase levels were increased by 1.4-fold after 2 weeks of RI (P<0.05). Creatinine and urea nitrogen levels were slightly increased by 26.5% (P<0.05) and 26.7% (P<0.05) respectively after 2 weeks of RI. After 2 weeks of operation, CCr in the model group was slightly decreased by 27.5% (P<0.05) compared with the sham group. Lactic acid, sodium, and potassium concentrations were not changed by RI. Renal function was still in the compensatory period. These results indicate successful establishment of an animal model of RI with mild renal insufficiency.

**Table 1:** Parameters associated with renal impairment and renal function.

	Sham group		Model group	
	Baseline	2 weeks	Baseline	2 weeks
Lactic dehydrogenase (U/L)	15.0±9.4	14.6±8.3	18.7±12.7	45.0±16.1^a^ ^b^
Lactic acid (mmol/L)	1.4±0.7	1.1±0.4	1.9±0.9	1.2±0.4
Creatinine (umol/L)	32.8±8.5	37.9±6.2	36.2±3.9	45.8±1.8^a^ ^b^
Creatinine clearance (ml/min/kg)		4.0±0.3		2.9±0.4^c^
Urea nitrogen (mmol/L)	2.2±0.4	2.8±0.7	3.1±1.0	4.2±1.0^a^ ^b^
Sodion (mmol/L)	148.7±2.6	151.6±1.1	148.4±3.1	149.0±3.2
Potassium (mmol/L)	5.1±0.4	4.7±0.1	4.5±0.5	4.6±0.3

a p<0.05 vs. baseline of model group;

b p<0.05 vs. 2 weeks of sham group;

c P<0.05 vs. 2 weeks of sham group. (mean ± standard deviation, n = 5).

### Effects of RI with Mild Renal Insufficiency on Heart Rate and P Wave Duration

**Figure 2:**
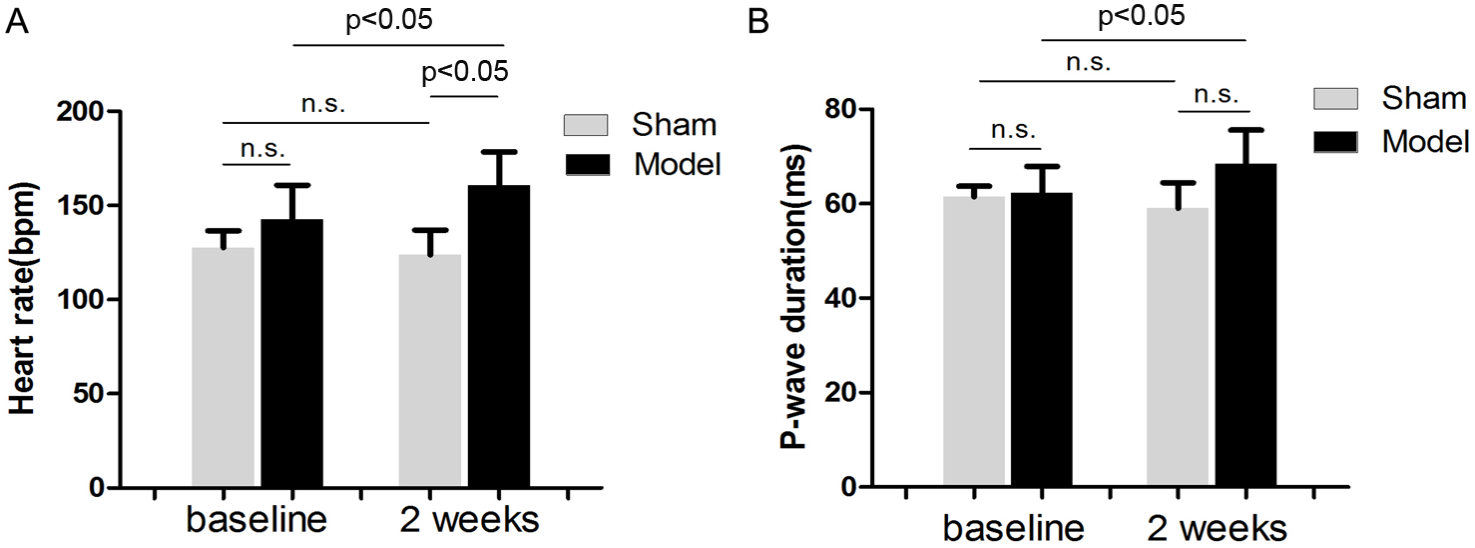
ECG analysis (n = 5). Effects of embolization versus sham operation on heart rate (A) and P-wave duration (B).

Figure 2 shows some ECG parameters in sham and model dogs during sinus rhythm. RI with mild renal insufficiency after 2 weeks of embolization resulted in a significant increase in heart rate (Figure 2A) by 12% (P<0.05), and prolonged the P wave duration (Figure 2B) by 12% (P<0.05) compared with baseline conditions in the model group. No changes were found in the sham group.

### Effects of RI with Mild Renal Insufficiency on BP and LV Pressure

**Figure 3:**
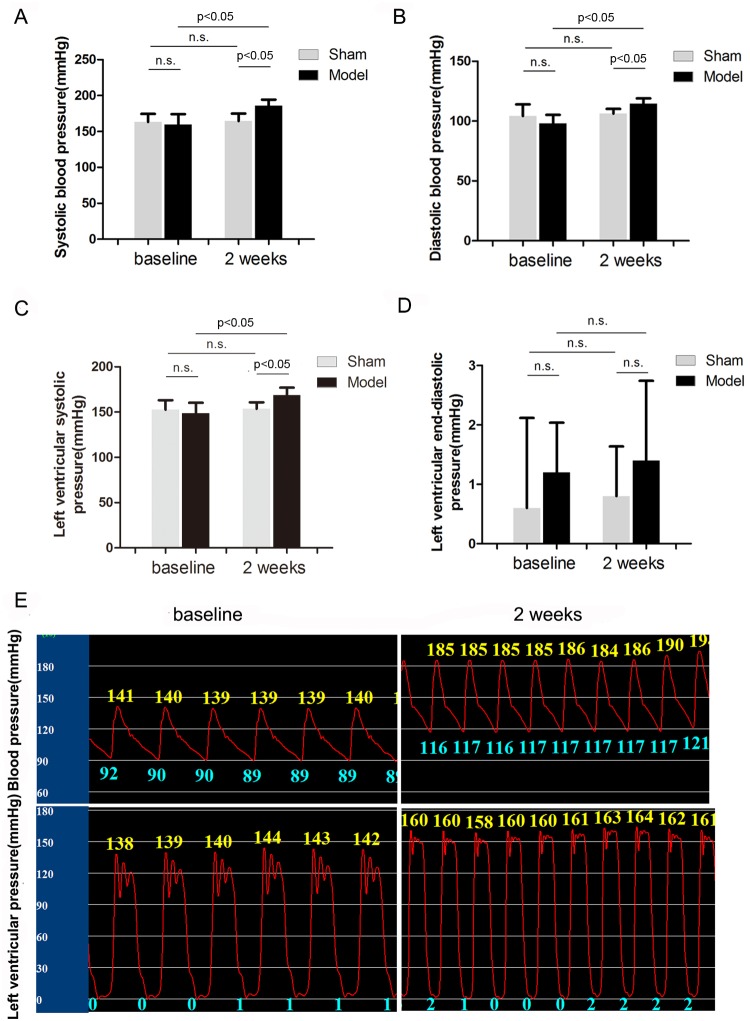
Analysis of BP and LV pressure (n = 5). Effects of embolization versus sham operation on SBP (A), DPB (B), LVSP (C) and LVEDP (D). Representative BP wave (upper) and LV pressure wave (lower) of dogs at baseline and with RI after 2 weeks of embolization in the model group (E).

Figure 3 shows BP and LV pressure in sham and model dogs during sinus rhythm. After 2 weeks of embolization, SBP (Figure 3A) was increased by 17% (P = 0.0032), DBP (Figure 3B) was increased by 16% (P<0.05), and LVSP (Figure 3C) was increased by 14% (P<0.05) compared with baseline values in the model group. However, LVEDP (figure 3D) was unchanged in the model group. No changes in these parameters were found in the sham group. Figure 3E shows a representative pressure wave of dogs at baseline and with RI after 2 weeks of embolization in the model group.

### Effects of RI with Mild Renal Insufficiency on Atrial Refractoriness

Effects of the sham procedure, embolization and rapid pacing on the AERP are shown in Table 2. In the model group, RI with mild renal insufficiency after 2 weeks of embolization resulted in a significant decrease in AERP by 10% (basic cycle length: 300 ms, P<0.05) and by 8% (basic cycle length: 240 ms, P<0.05) at two different stimulation frequencies compared with baseline values. The AERP after 2 weeks of sham operation was unchanged in the sham group. 30 minutes of rapid pacing resulted in a decrease of AERP (P<0.05) at baseline and after 2 weeks of intervention in both sham and model groups. There was no difference in the decrease of AERP after 30 minutes of rapid pacing between baseline and 2 weeks in sham and model group.

**Table 2:** Effects of embolization, sham operation and 30 minutes of rapid pacing on AERP.

		Baseline			2 weeks		
	BCL	Before pacing	After pacing	Decrease	Before pacing	After pacing	Decrease
Sham (ms)	300	144.0±17.5	136.0±14.7^a^	8.0±4.5	145.0±17.7	137.0±14.8^b^	8.0±4.5
	240	127.0±16.8	120.0±16.2^a^	7.0±2.7	127.0±14.8	120.0±15.4^b^	7.0±2.7
Model (ms)	300	149.0±12.0	142.0±10.4^a^	7.0±5.7	134.0±13.0^c^	122.0±11.5^b^ ^d^	12.0±8.4
	240	133.0±8.4	125.0±7.9^a^	9.0±4.2	122.0±5.7^c^	111.0±5.5^b^ ^d^	11.0±4.2

a p<0.05 vs. before pacing at baseline;

b p<0.05 vs. before pacing at 2 weeks;

c P<0.05 vs. before pacing at baseline;

d p<0.05 vs. after pacing at baseline. (mean ± standard deviation, n = 5).

### Effects of RI with Mild Renal Insufficiency on Inducibility and Duration of AF

**Figure 4:**
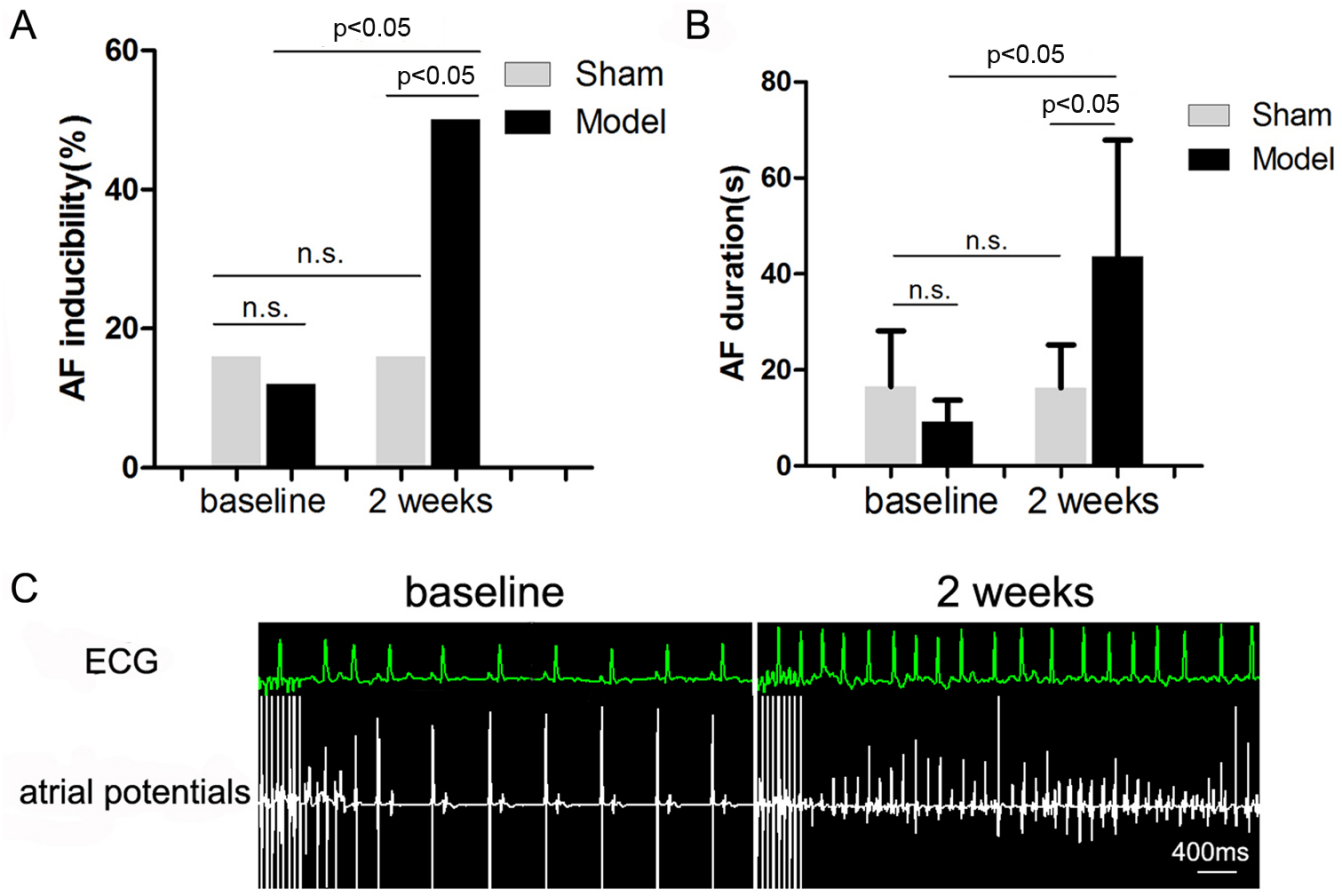
Effects of RI with mild renal insufficiency on the occurrence of AF (n = 5). Effects of embolization versus sham operation on inducibility of AF (A) and on the duration of induced AF episodes (B) in dogs. Representative right atrial potentials and ECG recordings after 10 seconds of rapid atrial pacing at baseline and with RI after 2 weeks of embolization in the model group (C).

RI with mild renal insufficiency after 2 weeks of embolization resulted in a significant increase in AF inducibility (Figure 4A) by 3.2-fold (P<0.05) and prolonged the duration of AF (Figure 4B) by 3.8-fold (P<0.05) compared with baseline values in the model group. The inducibility and duration of AF were unchanged in the sham group. Figure 4C shows representative right atrial potentials and ECG recordings after 10 seconds of rapid atrial pacing at baseline and with RI after 2 weeks of embolization in the model group.

### Effects of RI with Mild Renal Insufficiency on Antegrade Wenckbach Point, Atrial and Ventricular Rates during AF

**Figure 5:**
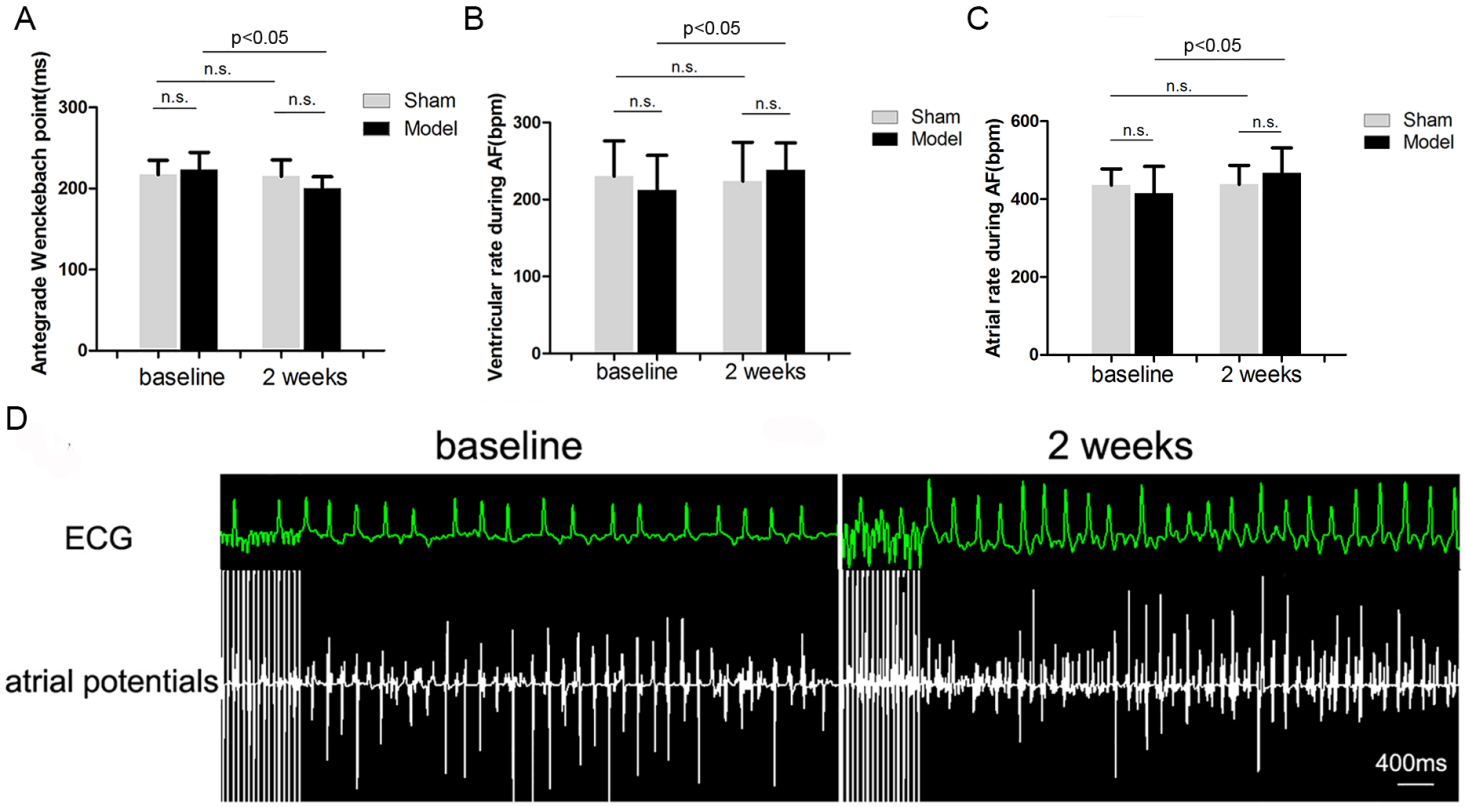
Electrophysiological effects of RI with mild renal insufficiency on AF and antegrade Wenckebach point (n = 5). Effects of embolization versus sham operation on antegrade Wenckebach point (A), ventricular rate during AF (B) and atrial rate during AF (C). Representative right atrial potentials and ECG recordings after 10 seconds of rapid atrial pacing at baseline and with RI after 2 weeks of embolization in the model group (D).

RI with mild renal insufficiency after 2 weeks of embolization resulted in a significant decrease in the antegrade Wenckebach point by 10% (P<0.05) (Figure 5A), an increase in ventricular rate during AF (Figure 5B) by 12% (P<0.05) and an increase in atrial rate during AF (Figure 5C) by 13% (P<0.05) compared with baseline values in the model group. Antegrade Wenckebach point, ventricular and atrial rates during AF were unchanged in the sham group. Figure 5D shows representative right atrial potentials and ECG recordings after 10 seconds of rapid atrial pacing at baseline and with RI after 2 weeks of embolization in the model group.

### Effects of RI with Mild Renal Insufficiency on the Systematic Activity of SNS, Inflammation and Oxidative Stress

**Figure 6:**
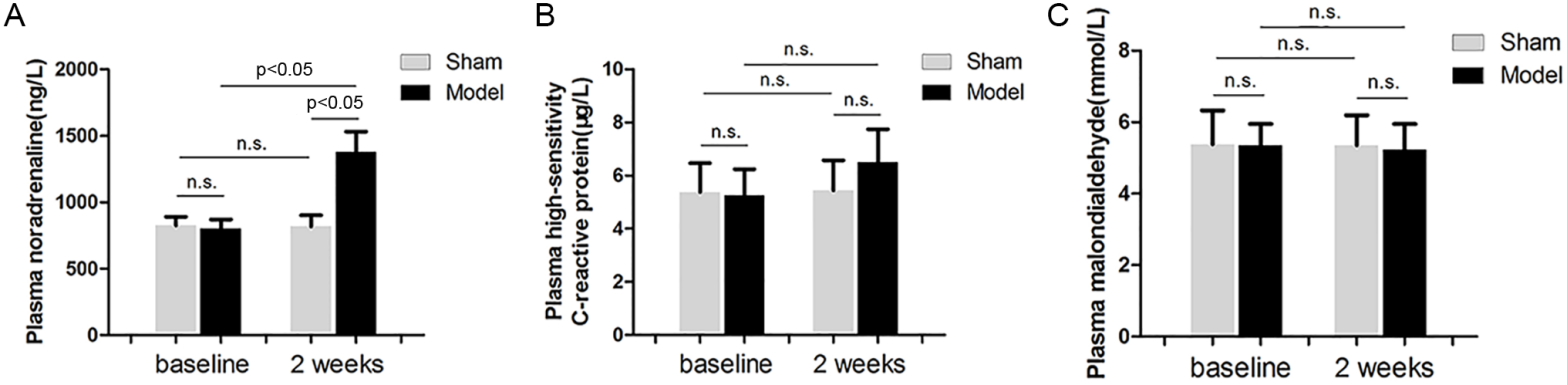
Effects of embolization versus sham operation on plasma noradrenaline (A), hs-CRP (B) and malondialdehyde (C) levels.

Plasma noradrenaline levels were measured to represent systematic activity of SNS. Plasma hs-CRP levels were measured to represent activity of systematic inflammation. Plasma malondialdehyde levels were measured for activity of systematic oxidative stress. RI with mild renal insufficiency after 2 weeks of embolization resulted in a significant increase in plasma noradrenaline levels (Figure 6A) by 72% (P<0.05) compared with baseline values in the model group. Plasma noradrenaline levels were unchanged in the sham group. There was a trend for an increase in plasma hs-CRP levels after 2 weeks of embolization in the model group, but no statistical significance was found. Overall, plasma hs-CRP (Figure 6B) and malondialdehyde (Figure 6C) levels were unchanged in the model and sham groups.

### Effects of RI with Mild Renal Insufficiency on RAAS

**Figure 7:**
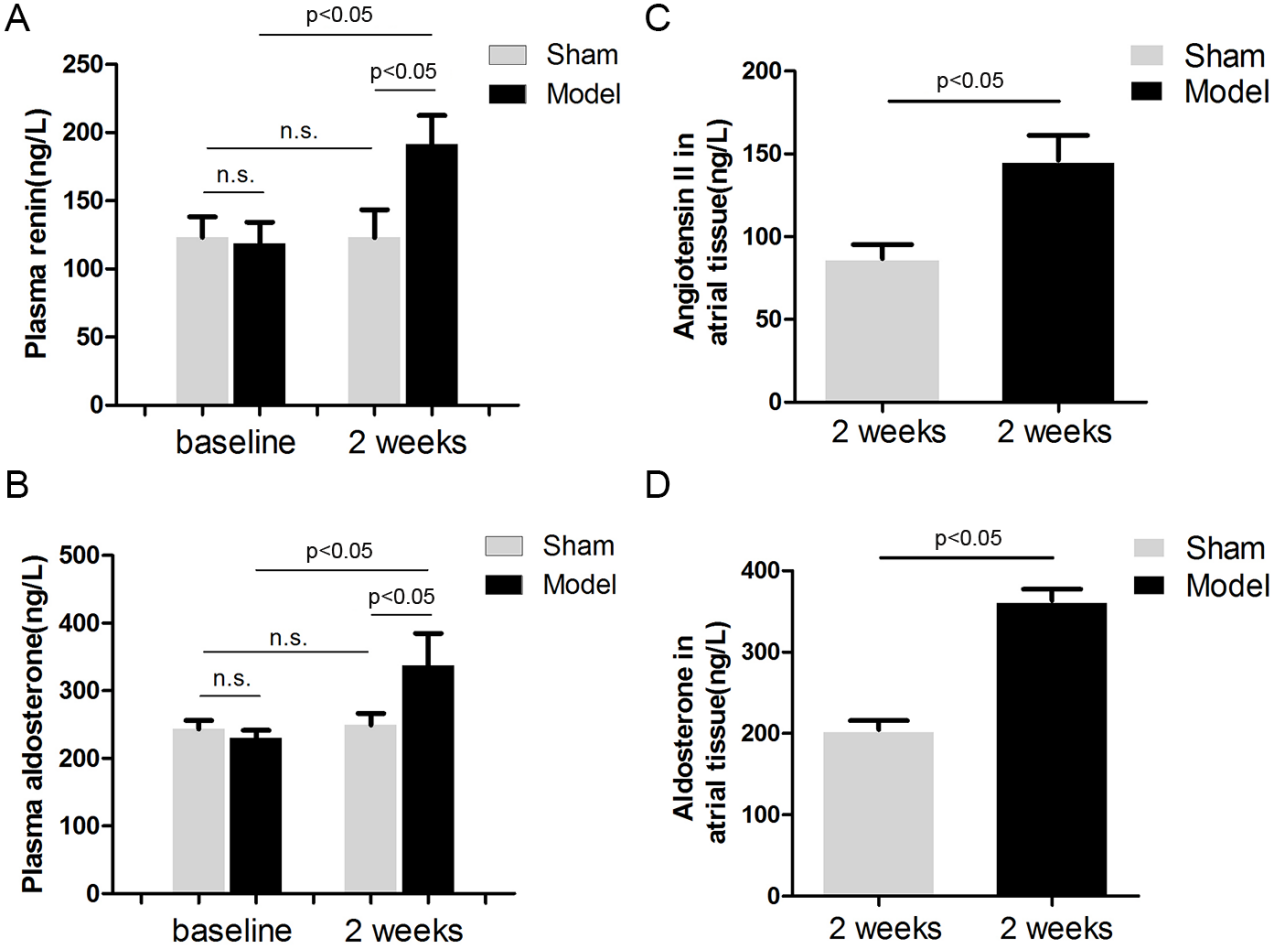
Effects of embolization versus sham operation on plasma renin (A) and aldosterone (B) levels, left atrial angiotensin II (C) and aldosterone (D) levels (n = 5).

Plasma rennin and aldosterone levels were measured to represent systematic activity of the RAAS. RI with mild renal insufficiency after 2 weeks of embolization resulted in a significant increase in plasma renin levels (Figure 7A) by 61% (P<0.05), and plasma aldosterone levels (Figure 7B) by 47% (P<0.05) compared with baseline values in the model group. Plasma renin and aldosterone levels were unchanged in the sham group. Left atrial tissue levels of angiotensin II (Figure 7C) and aldosterone (Figure 7D) were also elevated by 68% (P<0.05) and 77% (P<0.05) respectively in the model group, compared with the sham group.

### Effects of RI with Mild Renal Insufficiency on Atrial Fibrosis

**Figure 8:**
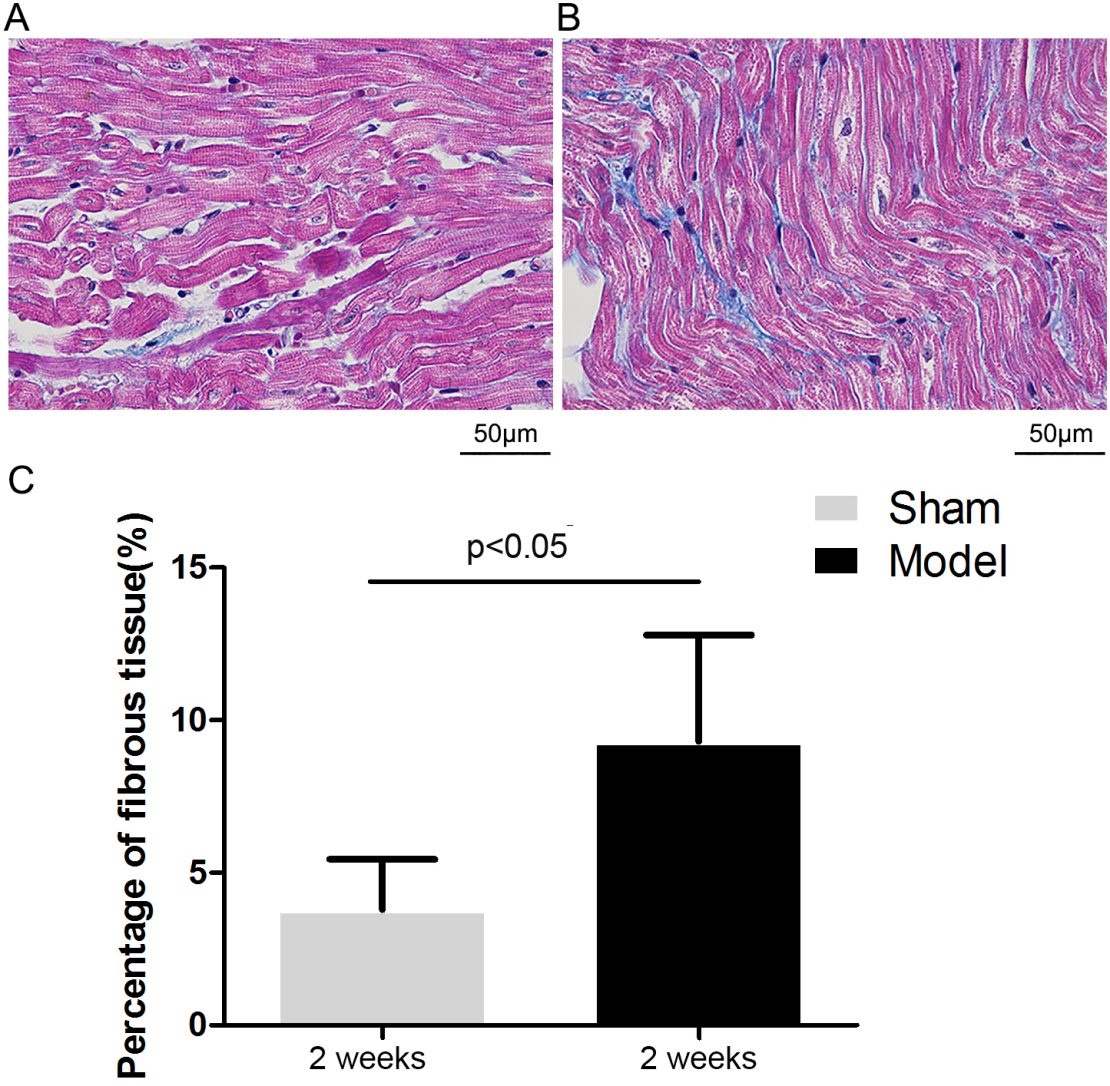
Analysis of atrial fibrosis. Representive images of Masson staining of the left atrial tissue after 2 weeks of interventional operation in the sham (A) and model groups (B). Mean percentage of interstitial fibrosis of the left atrium (C).

Figure 8A and 8B illustrates representive images of Masson staining of the left atrial tissue after 2 weeks of interventional operation in the sham and model groups, respectively. The quantitative ratio of the area of interstitial fibrosis was summarized in Figure 8C. Compared with the Sham group (3.8%±1.6%), extensive and heterogeneous interstitial fibrosis was observed in the model group (9.3% ±3.5%, P<0.05).

## Discussion

The main findings of our study were: 1) embolization of small renal artery branches of the left kidney for 2 weeks resulted in ischemic RI with mild renal insufficiency; 2) RI with mild renal insufficiency was associated with vulnerability to AF; 3) increased vulnerability of AF might be associated with increased activity of the SNS, RAAS, and atrial fibrosis in the model of RI with mild renal insufficiency.

To date, there has only been one study [9] on rats showing that CKD is associated with the development of AF. In this previous study, a classical model of CKD was created in rats with 5/6 nephrectomy. Oxidative stress may have been involved in the pathogenesis of interstitial fibrosis and enhanced vulnerability to AF in the left atrium in this CKD model. Besides the mechanism of oxidative stress, inflammation, the RAAS, and SNS activation are predicted to play important roles in the development of AF associated with CKD [10].

We established a new *in* *vivo* model of RI in a large animal (dogs) and found that ischemic RI with mild renal insufficiency was associated with vulnerability to AF. In Fukunaga et al’s study [9], a stage 4 CKD model was created by 5/6 nephrectomy, indicating that renal function was severely damaged. In our study, unilateral diffuse ischemic RI was induced in dogs by transcatheter embolization of small renal artery branches using gelatin sponge granules. This method was simple and did not produce severe trauma in the dogs compared with 5/6 nephrectomy, and it did not cause whole organ infarction or severe renal dysfunction. Wang and Bao [11] found that renal function did not significantly change, even after 1 month of unilateral nephrectomy, indicating that the unilateral kidney could undertake compensatory function. In our study, creatinine and urea nitrogen levels were slightly increased, CCr was slightly decreased, which might be associated with the effects of persistent and unilateral RI on the normal contralateral kidney [12]. Factors induced by severe renal dysfunction were eliminated in our study because renal function in our study was still at the stage of the compensatory period.

Effects of hypertension on left atrial pressure and vulnerability to AF were not present in our study. Hypertension can be induced by severe renal dysfunction, and also by activation of the RAAS and SNS induced by RI [13]. Long-term hypertension is associated with high atrial pressure and atrial enlargement predisposing to AF [7]. LV systolic and diastolic function was not investigated in Fukunaga et al’s study [9]. We found that although BP and LVSP were significantly elevated after 2 weeks of RI, LVEDP was not changed. This finding indicated that hypertension did not affect left atrial pressure and its effect on vulnerability to AF was negligible. A possible reason for this finding may be because the length of time of hypertension was too short to affect left atrial pressure. Overactivity of the RAAS and SNS could contribute to elevated BP and LVSP in our model.

Heart rate was significantly increased in our study. Heart rate had a tendency to rise in a CKD model, but this was not significant in Fukunaga et al’s study [9]. Ye et al [12] found that renal injury caused by phenol injection significantly increased heart rate and BP, which persisted for more than 3 weeks. Another study showed a significant increase in heart rate in a model of CKD, which was created in rats with ¾ nephrectomy. Heart rate does not significantly increase in CKD patients [14], which may be associated with the baroreflex. Renal status may affect the distribution of baroreflex and nonbaroreflex activity, as well as the strength of the SBP-heart rate relationship [15]. Several studies have also found that end-stage renal disease patients have a withdrawal in parasympathetic modulation of heart rate in conjunction with an increase in sympathetic input to the sino-atrial node [16]. Activity of the SNS and sensitivity of the baroreflex might have affected heart rate in our study.

More electrophysiological parameters can be detected in large animal models than small animal models. P wave duration, and episodes and duration of AF were increased after RI compared with baseline in our study, which is consistent with Fukunaga et al’s study [9]. Interstitial fibrosis might have led to prolongation of P wave duration in our study. Additionally, we found that the atrial and ventricular rates during AF were increased, and the antegrade Wenckebach point was shortened by RI with mild renal insufficiency. The increased atrial rate during AF could be the result of shortening of the effective refractory period. The increased ventricular rate during AF could be the result of shortening of the antegrade Wenckebach point, which might be induced by overactivity of the SNS. More attention should be paid to the increased ventricular rate during AF in this model in the future, because control of this rate is important for patients with AF in clinical practice.

The design of our study is closer to the real clinical situation than other previous models. In the research field of AF, large animals, such as pigs and canines, are the most commonly used, because rats are not ideal for electrophysiological studies and catheter operations because of their fast heart rate and small size. In Fukunaga et al’s study [9], hearts had to be isolated, and electrophysiological parameters and vulnerability to AF had to be detected *in* *vitro* because rats were used, whereas electrophysiological parameters and vulnerability to AF could be detected *in* *vivo* in dogs in our study.

Individual differences in electrophysiological parameters are always large. Therefore, we designed a before–after study to reduce the effect of individual differences, because large animals can be conveniently and repeatedly monitored. In Fukunaga et al’s study, no significant differences were observed in the effective refractory period of the left atrium between the sham and model groups [9]. The effective refractory period of the right atrium also showed no significant difference between the sham and model groups in our study, whereas a significant difference was observed between baseline and 2 weeks in the model group. Overactivity of the SNS might play an important role in shortening of the effective refractory period because just adrenergic stimulation can decrease the human AERP by approximately 5% [17].

Our model was suitable for further determining the predominant factors that enhance AF vulnerability. Predicted mechanisms for the development of AF associated with CKD were observed in our study, including RAAS, SNS activation and atrial fibrosis. There was also a trend for an increase in plasma hs-CRP levels in the model group, but this was not significant. CRP, as a marker of inflammation, is elevated in chronic renal impairment [18]. Serum CRP concentrations are positively correlated with AF persistence, and predict postoperative AF occurrence [19]. The RAAS is activated by renal ischemic impairment and increased sympathetic activation in CKD [20]. The RAAS is involved in the pathogenesis of interstitial fibrosis, and they create a substrate for AF [21], [22], [23]. The injured kidney’s afferent signals to central integrative structures in the brain lead to increased sympathetic activation [24]. Chemoreflex activation, reduced nitric oxide availability, and renalase secretion are also involved in heightened sympathetic tone and increased noradrenaline levels in patients with kidney impairment [24]. Increased sympathetic activation is found in the initial clinical stages of CKD [25], which also leads to atrial remodeling processes, possibly by neurohumoral activation and changes in atrial hemodynamics [26]. Hyper-sympathetic activity may facilitate the initiation of AF and acute atrial electrophysiological changes [27]. Understanding these mechanisms could lead to new therapeutic strategies for CKD patients combined with AF. Renal denervation, which is a new therapeutic approach to treat resistant hypertension through reducing renal norepinephrine spillover, can also prolong the antegrade Wenckebach point, and provides control of the ventricular rate during AF in normal pigs [28]. Renal denervation also inhibits pronounced shortening of the AERP and reduces susceptibility to AF in a pig model of obstructive sleep apnea or heart failure [29], [30] by combined reduction of sympathetic drive and RAAS activity [31], [32]. Whether this treatment has the same effects in our canine model of RI with mild renal insufficiency may be important for elucidating mechanisms and developing new therapeutic strategies for CKD-induced hypertension and atrial arrhythmogenic remodeling.

### Study Limitations

The pathophysiological process and severity of renal impairment in our model is not completely in accord with the real situation of CKD. AF mainly originates from the left atrium, but AF was induced in the right atrium in our study because the left atrium is difficult to reach through a catheter operation. Spontaneous induction of AF is too rare for systematic evaluation. Therefore, we applied fast pacing to induce AF (AF begets AF). The sensitivity of the baroreflex and the strength of sympathetic and parasympathetic modulation of the sino-atrial node in the baroreflex may influence AF vulnerability. Nonetheless, these effects could not be eliminated. Intervention measures, such as renal denervation, administration of angiotensin-converting enzyme inhibitors or β-adrenoceptor blockers need to be applied for further clarifying the related mechanisms.

## Conclusions

We successfully established an *in* *vivo* model of RI with mild renal insufficiency in a large animal and showed that AF was associated with RI with mild renal insufficiency in this model. Increased activity of the SNS, RAAS and enhanced atrial fibrosis may contribute to the development of AF associated with RI with mild renal insufficiency. A successful model of RI with mild renal insufficiency in canines could be used to further investigate the factors responsible for the development of AF associated with CKD in the future.
